# Treatment response evaluation in an *ex vivo* model of *E. coli*-infected central venous catheter system

**DOI:** 10.3389/fped.2025.1421992

**Published:** 2025-06-18

**Authors:** Zihe Huo, Corinne Légeret, Stefan G. Holland-Cunz, Stephanie J. Gros

**Affiliations:** ^1^ Department of Pediatric Surgery, University Children's Hospital Basel, Basel, Switzerland; ^2^ Department of Clinical Research, University of Basel, Basel, Switzerland; ^3^ Department of Pediatric Gastroenterology, University Children's Hospital Basel, Basel, Switzerland

**Keywords:** central venous catheter, infection, treatment, antibiotics, microcalorimeter, parenteral nutrition, isothermal micorcalorimetry

## Abstract

**Introduction:**

Despite all precautions, central line-associated bloodstream infections (CLABSI) are inevitable, especially in children. Different treatment strategies exist for those situations. This study aims to compare the different treatment strategies.

**Methods:**

In this study, central venous catheters (CVC, Broviac single lumen) were contaminated with *E. coli in vitro*. Different treatments (70% ethanol, ceftriaxone, TauroLock) were applied, and the effect was measured by isothermal microcalorimetry.

**Results:**

A rapid decrease in heat release corresponds to a rapid decrease in the number of living bacteria. Ceftriaxone had the quickest effect followed by ethanol in combination with ceftriaxone, ethanol, and TauroLock.

**Discussion:**

Antibiotics must be based on patient risk factors, the severity of infection, and local resistance pattern; therefore, it is difficult to publish general guidelines applying to all children. In this *in vitro* study, ceftriaxone demonstrated the most the highest efficacy on the bacteria. Taurolidine locks are recommended for preventing CLABSI, but no data are available in regards using it for treatment. In this setting, it was efficient, as was ethanol. However, the bacteria used in this study, have not been exposed to antibiotics before—this is most likely in contrast to patients, who have a central venous catheter.

**Conclusion:**

Under *in vitro* conditions, systemic ceftriaxone is the most efficient and fastest treatment for an *E. coli*-infected CVC. Elimination of bacteria was also reached with 70% ethanol and TauroLock, but it needed more time.

## 1 Introduction

Central venous catheters (CVC) have revolutionized the care and clinical outcomes of patients requiring long-term venous access. It is widely used among most pediatric health disciplines. Various treatments can be administered ranging, for example, from antibiotics for chronic osteomyelitis to chemotherapy in oncologic patients, to lifelong parenteral nutrition for children with gastrointestinal failure.

Intestinal failure is characterized by the reduction of functional intestinal mass below that which can sustain life, resulting in dependence on parenteral support for a minimum of 60 days within a 74 consecutive day interval (1). Thus, children with intestinal failure depend on central venous access to survive for the rest of their lives. In these children, central line-associated bloodstream infections (CLABSI) continue to be a significant cause of mortality, morbidity, and increased costs for affected patients and/or the health system. A German group calculated an incidence of 10.6 cases of CLABSI per 1,000 CVC days causing hospital costs directly attributed to it of 8,810 €/case (2). In most studies, *Escherichia coli* (*E. coli*) belongs to the most frequent originator of catheter infections, especially in small children (3, 4). As preventive measures, several published guidelines have been developed, providing comprehensive recommendations for the prevention of CLABSI including general hygienic measures (5). But despite all precautions, infections are inevitable, especially in children requiring home parenteral nutrition (6). These children usually can participate in normal life including school and extracurricular activities. As venous access sites are limited and are needed throughout life, several catheter salvage strategies have been established to save an existing catheter despite infection, even if only temporarily (7). There is, however, an ongoing discussion about which salvage strategy is best. The North American Society of Gastroenterology (NASPGHAN) recommends treatment with ceftriaxone in cases of suspected CLABSI (6). The latest recommendations of the European Society for Clinical Nutrition (ESPEN) from 2018 are less specific but state that the antibiotic should cover gram-positive staphylococci and gram-negative bacilli (8). Among the rescue strategies are the use of ethanol locks, antibiotic locks, and taurolidine locks, often combined with intravenous antibiotic treatment. Within our clinic, these treatment strategies are implemented based on the preferences of the pediatric subspecialties. Conducting a prospective clinical trial to evaluate these rescue strategies is currently not feasible due to the limited data available on children.

Isothermal microcalorimetry is a method that allows real-time monitoring of the metabolic activity of living organisms under variable conditions. Previous applications include the investigation of different aggregates, probes, and organisms. In this context, isothermal microcalorimetry has been used for the detection of bacterial infection, drug sensitivity testing, and drug screening in microbiology and food microbiology, as well as for material testing, monitoring, and parasitological applications (9–13). Measurements are taken in the range of microwatt, or lower, under strict isothermal conditions (14). Modern microcalorimeters reach a much higher sensitivity compared with more conventional methods such as spectrophotometry or enzymatic assays. The metabolic heat production of 10^4^–10^5^ bacteria, 10^3^–10^4^ protozoan, or 10^3^–10^4^ hepatocytes can also be detected in stool samples of mice (15, 16). The use of isothermal microcalorimetry measurements to evaluate treatment response and metabolic activity in tumor cells and tumor slice cultures has recently been described by our group (17–20). Isothermal microcalorimetry can be used to detect bacterial growth as well as respond to antibacterial drugs with very high sensitivity.

In our study, we investigate the effectiveness of the different treatment options that would regularly be used for CLABSI in children requiring using a Broviac catheter system that is routinely used in our clinic. We use an *ex vivo* contamination model and evaluate bacterial growth and response to antibacterial drugs using isothermal microcalorimetry.

## 2 Materials and methods

In- and exclusion criteria do not apply, as it is an *in vitro* study, and the desired conditions are produced. The procedure is reported in detail below.

### 2.1 Bacterial culture

*E. coli* [genotype: F-mcrA Δ(mrr-hsdRMS-mcrBC) φ80lacZΔM15 ΔlacX74 recA1 araD139 Δ (ara- leu)7697 galU galK rpsL (StrR) endA1 nupG] was thawed and cultured for 12 h with shaking at 200 rpm/min at 37°C. A central venous catheter (Broviac 6.6 French single-lumen CV Catheter, 0600540CE, Bard Access Systems, Inc., Salt Lake City, UT, USA) was incubated with the *E. coli* culture overnight. New, unwashed catheters were cut into 1 cm segments. Roswell Park Memorial Institute (RPMI) 1640 medium (Sigma-Aldrich, Munich, Germany) containing biotin and vitamin B12 was used, supplemented with 10% fetal calf serum (FCS) as it has been found suitable for a variety of mammalian cells (21). We added human whole blood from healthy donors, which was collected in ethylenediaminetetraacetic acid (EDTA) tubes, directly to the catheter pieces, and added medium to determine the condition that most realistically portrayed the situation in the patient. In the next step, catheters were treated with different therapeutic regimens that were selected based on the guidelines from the North American Society of Pediatric Gastroenterology Hepatology and Nutrition (NASPGHAN) (6): 70% ethanol in medium (Sigma-Aldrich, Munich, Germany), ceftriaxone at a dose of 80 mg/kg of body weight in medium (Roche, Rotkreuz, Switzerland), taurolidine, and 4% citrate (TauroPharm GmbH, Germany) (*n* = 4 per group) before preparing them for microcalorimetric measurements. A negative control group was set up with catheters in sterilized phosphate-buffered saline (PBS) without bacterial incubation to show how much heat a sterile catheter produces, as normal control.

### 2.2 Isothermal microcalorimetry

For isothermal microcalorimetric measurements, a 48-channel isothermal microcalorimeter (calScreener, Symcel AB, Stockholm, Sweden) was used as previously described (22). A correlation between bacterial activity and thermal development has been shown in a previous study (11). The vials were then sealed and inserted in the well-plate microcalorimeter according to the manufacturer’s instructions. For optimal performance, multiple separate reference vessels were included. Each reference vessel was filled with an inert sample (medium only), which was used as a thermal reference. Following that, thermal equilibration measurements were recorded with the thermostat set at 37°C to minimize sources of error. The microcalorimetry data were sampled at a frequency of one data point every 60 s for 250 h until the metabolic heat signal returned to baseline. The heat release of *E. coli* in different conditions and with different antibacterial drugs was measured for 80 h.

The experiment was then repeated. Data were stored by Symcel calView software and exported as a CSV file that could be edited in commonly used spreadsheet software. Data were analyzed using GraphPad Prism 8.4 software. The higher the curve, the more bacteria are alive releasing heat.

## 3 Results

### 3.1 Experimental conditions

**Figure 1:**
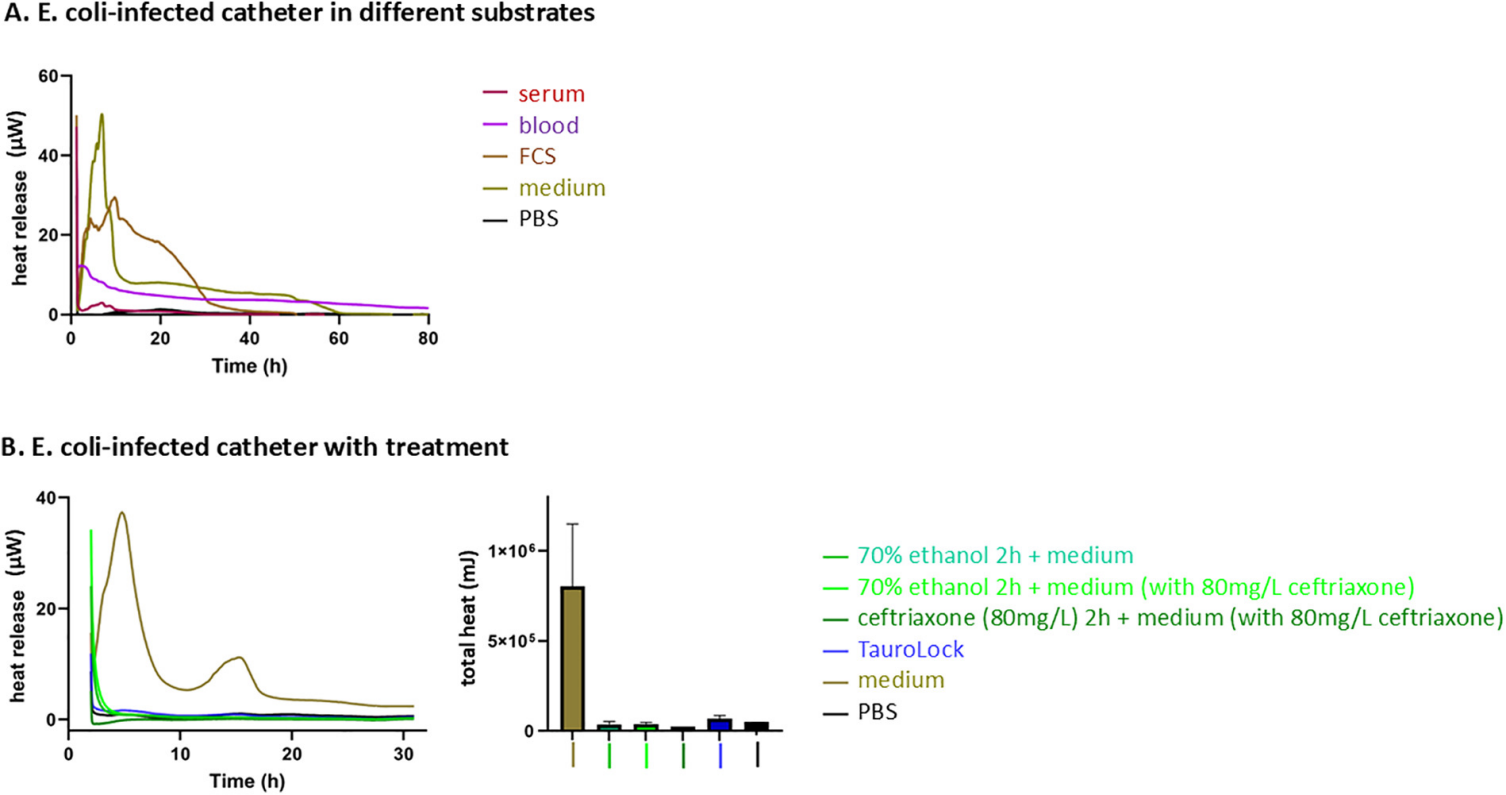
*E. coli*-infected catheter in different substrates and with different treatments. ** (A)** The heat release curve of *E. coli* in different substrates. PBS without bacteria serves as a control. *E. coli* in medium (RPMI1640 with 10% FCS) shows a typical heat release curve with two peaks as has previously been shown (20). Using human or fetal calf blood components leads to a decrease in *E. coli* heat release, most likely due to an immune response. **(B)** The heat release curve and the total heat curve of *E. coli* in the medium with respect to the four treatment options. PBS without *E. coli* serves as a negative control. The fastest and strongest response in heat decline can be observed for treatment with ceftriaxone followed by taurolidine, while the curves of ethanol lock alone and ethanol lock with ceftriaxone lead to a slower decrease in *E. coli* activity. Activities eventually seize completely, suggesting bacterial death. All figures show the heat release curve of bacteria—the higher the curve, the more heat is released, which in turn corresponds to more bacteria. Accordingly, a zero line corresponds to a sterile liquid.

Firstly, we wanted to determine the best conditions for investigating *E. coli* catheter infection *ex vivo*. Obviously, it would be ideal to perform these experiments in catheters surrounded by patients’ blood. When adding blood from the immunocompetent donor, we observed a suppression of *E. coli* activity in the culture, whereas cultivating *E. coli* in RPMI 1640 resulted in the typical heat release curve previously reported for *E. coli* (Figure 1A) (20). RPMI 1640 medium was chosen as it is superior to the Luria–Bertani (LB) medium for mimicking the human blood physiological salt and pH conditions. The LB medium only serves as a growth medium for bacteria. Therefore, the RPMI 1640 medium was used to perform therapeutic catheter experiments. PBS alone served as a negative and sterility control and showed no thermal activity.

### 3.2 Treatment

When testing the catheters that had been infected with *E. coli*, we used a choice of clinically used, recommended, and still highly controversially discussed therapeutic regimens (Figure 1B). The most rapid decrease in *E. coli* activity was observed under treatment with ceftriaxone corresponding to the fastest response to the antibiotic well within 6 h. A rapid decrease in heat release over time corresponds to a rapid decrease in the number of living bacteria (Figure 1B, first graph). The second most efficient treatment option was treatment with taurolidine, followed by an ethanol lock alone and accompanied by ceftriaxone. Differences between the treatment groups could also be demonstrated by the total heat measurements (Figure 1B, second graph). By using isothermal microcalorimetry, drug response can be assessed within 6–12 h of treatment.

## 4 Discussion

Despite the limitations of these experimental *ex vivo* conditions, our study shows that antibiotic treatment is the most effective in case of contamination with *E. coli* without prior resistance to antibiotics followed by taurolidine. The antibacterial response was delayed using ethanol locks followed by regular medium or treatment with ceftriaxone but efficient.

In our experimental setup, we sought to generate the conditions of the liquid compartment (human blood) to mimic catheter infection as close as possible to the situation in the child. However, we encountered some challenges. When using the immunocompetent blood of healthy volunteers, it quickly became obvious that the natural immune response of the competent donors led to a quick, spontaneous decline of *E. coli* activity *in vitro*. Similar results were obtained when using fetal calf serum. Instead of using LB medium, which would have been the most logical choice of medium to culture *E. coli* bacteria, we used RPMI1640. This much more closely imitates the physiological conditions of the human blood especially regarding salt content and pH without providing immune system components (15). This was highly important for us in this experimental setting as children in need of CLABSI often suffer from an underlying hematological, oncological, or immunological disease and are mostly undernourished and therefore, to different extents, not immunocompetent. Moreover, using RPMI1640 resulted in a heat release curve typical for monitoring *E. coli* by isothermal microcalorimetry and could therefore be well compared.

The choice of antibiotics for each patient obviously depends on individual patient risk factors, the severity of the infection, and prior antibiotic resistance. Resistance to antibiotics is an emerging threat for children, and even *E. coli* strains from neonates can be found to be ceftriaxone-resistant (23, 24). In our study, we used ceftriaxone, a parenteral cephalosporin with a half-life of 8 h which is widely used in pediatric patients of all ages and has a good safety profile (25). Therefore, it is recommended in cases of suspected CLABSI by the North American Society of Gastroenterology (NASPGHAN) (6). The European Society for Clinical Nutrition (ESPEN) guidelines on treatment for CLABSI keep the recommendations less specific, stating “the choice of empirical antibiotic therapy for CLABSI should usually include coverage for gram-positive coagulase-negative or -positive staphylococci and gram-negative bacilli” (8). Clearly, antibiotics must be based on patient risk factors, the severity of infection, and local resistance pattern; therefore, it is difficult to publish general guidelines applying to all children except for infections of long-term catheters with *S. aureus*, *P. aeruginosa*, mycobacteria, or fungi. In this case, the Infectious Disease Society of America recommends the prompt removal of the catheter (26). The guidelines of this society also recommend the empirical coverage for gram-negative bacilli (including *E. coli*) to be a cephalosporin, carbapenem, or β-lactam/β-lactamase combination with or without an aminoglycoside (26). Microorganisms can not only cause acute infections, but they can also colonize medical devices and get organized in biofilms. Bacteria in biofilms persist by a strategy of tenacious survival rather than aggressive virulence. Biofilm infections can linger for months or even years, rarely being fatal but often sitting undisturbed by antibiotic treatment. Treatment of infections caused by colonization of biofilms often fails, as these infections require higher antibiotic dosages for a prolonged time (27). Taurolidine lock is known to be effective in preventing catheter-related infections in a variety of venous access devices (28). Literature about the use of taurolidine for treating catheter colonization or CLABSI is sparse. One study including 24 patients with CLABSI was found in which taurolidine was used in combination with systemic antibiotic therapy and was successful in treating all cases (29). However, clinical studies in which patients with CLABSI have only been treated with taurolidine are lacking. Most probably because the probationary treatment of a systemic disease with a local treatment is ethically not justifiable. Due to the vague data availability, the Infectious Disease Society of America recommends that taurolidine should only be used for catheter salvage (26). Interestingly, in our experimental study setup, taurolidine lock proved to be efficient. The ESPEN states that antibiotic line locks are effective in preventing CLABSI and should be used during long-term catheter use but should not be the only treatment for treating CLABSI, as there are no data on the effectiveness of single lock therapies. They can however be used in conjunction with systemic antibiotics (30). Ethanol is an antiseptic and has in a meta-analysis of studies comparing heparin, and ethanol locks been proven to reduce the risk of CLABSI by 81% and the need for CVC replacement by 72% (31). The ESPEN advocates ethanol line locks to be considered for preventing CLABSI (8). Wolf et al. (32) assessed the effect of ethanol not for prophylaxis, but for treatment of CLABSI. A total of 94 pediatric cancer patients aged 6 months to 24 years with CLABSI were randomly assigned to either receive ethanol lock therapy with 70% ethanol or placebo for 2 h daily for 5 days. Treatment failure was observed in 44% and 43%, respectively, in the groups. However, catheter occlusion requiring thrombolytic therapy was more commonly seen in catheters treated with ethanol lock therapy (58%) than with placebo (33%). In our *ex vivo* experiment, antibacterial response using ethanol lock was delayed compared with antibiotic treatment only but efficient.

A glimpse at the latest literature regarding CLABSI in pediatrics shows that an increase in the United States from 2019 to 2022, compared with 2016–2019 (33), was noted. Most current literature (34, 35) refers to the prevention of CLABSI, which involves a bedside tool to identify patients at greatest risk for a line infection (including frequency of dressing changes, skin at entry sites, etc.), the teaching of nurses and family, and overall awareness. Only one study (36) focuses on the treatment of CLABSI: taurolidine lock as used in addition to antibiotic treatment with the aim of catheter salvage in neonates with difficult vascular access. This strategy was successful in 86%, and no major adverse events were noted.

A major advantage of using isothermal microcalorimetry to evaluate treatment response is the real-time monitoring of bacterial heat reduction. In our case, treatment response could be determined within 6 h. Our vision for the future is that the patient's blood, rather than catheters—after enrichment with a medium to ensure bacterial detection—will be examined directly using microcalorimetry to measure response to a choice of antibacterial treatments individualized for each patient. Similar already established systems (e.g., BACTEC) allow enrichment and real-time detection of bacteria in blood cultures. This would also take the patient’s own immune components into account. Using isothermal microcalorimetry for drug-response assessment can refine the choice of treatment for CLABSI in children significantly in the future. The disadvantages of this study are, on one hand, that we only tested one group of bacteria, which had not yet come into contact with antibiotics and therefore cannot be resistant, and on the other hand, the nature of the study, namely, that it is an *in vitro* model.

Our data clearly show an advantage of using ceftriaxone for treatment in our *ex vivo* experimental setting. Clinical use of the described treatments is still controversial and applied differently in different parts of the world. Based on our findings, a prospective clinical trial should be implemented for rescue therapies of CLABSI when removal of the catheter is not an easy option.

## 5 Conclusion

Under *ex vivo* conditions, ceftriaxone is the most efficient and fastest treatment for an *E. coli*-contaminated CVC followed by taurolidine. The antibacterial response was delayed using ethanol locks followed by regular medium or treatment with ceftriaxone but efficient. Using isothermal microcalorimetry for drug-response assessment can refine the choice of treatment for CLABSI in children significantly in the future by continuously measuring the response to the antibiotic. However, this requires standardized protocols for the clinical use of isothermal microcalorimetry. Based on our findings, a clinical trial could be implemented to evaluate catheter rescue strategies in children.

## Data Availability

The original contributions presented in the study are included in the article/Supplementary Material, further inquiries can be directed to the corresponding author.
