# Supplementary figures and images for: Treatment response evaluation in an ex vivo model of E. coli-infected central venous catheter system
Source: Front Pediatr. 2025 Jun 18;13:1421992. doi: 10.3389/fped.2025.1421992 (PMC12213736; doi:10.3389/fped.2025.1421992)

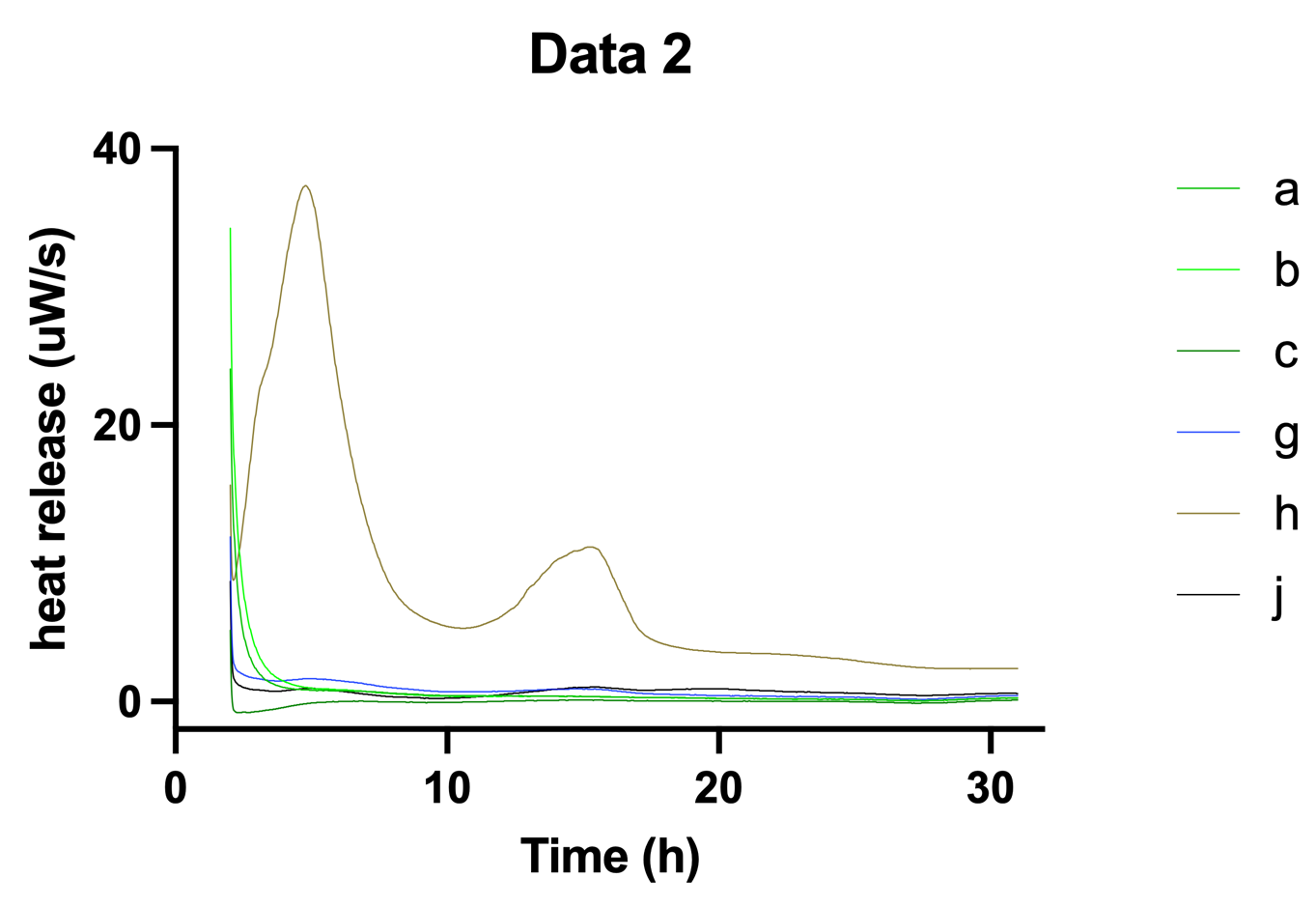

Supplement: Supplementary file 1 [file Image1.tiff]
